# Community-Associated Methicillin-Resistant *Staphylococcus aureus* Among Gay, Bisexual, and Other Men Who Have Sex with Men Living with HIV Who Engage in Chemsex in Barcelona, Spain: Epidemiological Patterns and Implications for Infection Control

**DOI:** 10.3390/microorganisms14081752

**Published:** 2026-08-09

**Authors:** Giulia Teodora Cosmulescu Șpaiuc, Joan Gil Miñana, Lorena de la Mora, Cristina Pitart, Leire Berrocal, Elisa de Lazzari, Montserrat Laguno, Berta Torres, Ana González-Cordón, Berta Isabel Fidalgo, Mariana José Fernández, Lucía González, Alexandra Calmy, Laura Morata, Alexy Inciarte, Alberto Foncillas, Julia Calvo, Paula Arreba, Pilar Callau, Estela Solbes, Abiu Sempere, Ivan Chivite, José Luis Blanco, Juan Ambrosioni, Esteban Martínez, Alex Soriano, Josep Mallolas, Maria Martínez Rebollar

**Affiliations:** 1 Campus Clínic, Faculty of Medicine, University of Barcelona, 08036 Barcelona, Spain; 2 HIV Unit, Infectious Diseases Service, Hospital Clínic, AIDS and HIV Research Group, Fundació de Recerca Clínic Barcelona–Institut d’Investigacions Biomèdiques August Pi i Sunyer (IDIBAPS), University of Barcelona, 08036 Barcelona, Spain; 3 Institute of Global Health, Faculty of Medicine, University of Geneva, 1202 Geneva, Switzerland; 4 Department of Clinical Microbiology, ISGlobal, Hospital Clínic, University of Barcelona, Centro de Investigación Biomédica en Red de Enfermedades Infecciosas (CIBERINFEC), 08036 Barcelona, Spain; 5 Centro de Investigación Biomédica en Red de Enfermedades Infecciosas (CIBERINFEC), Instituto de Salud Carlos III, 28029 Madrid, Spain; 6 Global Tuberculosis Program, Department of Migrant Health, Baylor College of Medicine, Houston, TX 77030, USA; 7 HIV Unit, Infectious Disease Division, Department of Medicine, Geneva University Hospitals, 1205 Geneva, Switzerland; 8 Infectious Diseases Service, Hospital Clínic, Fundació de Recerca Clínic Barcelona–Institut d’Investigacions Biomèdiques August Pi i Sunyer (IDIBAPS), University of Barcelona, 08036 Barcelona, Spain

**Keywords:** community-associated methicillin-resistant *Staphylococcus aureus*, chemsex, HIV, antimicrobial resistance, gay, bisexual, and other men who have sex with men

## Abstract

Background: Community-associated methicillin-resistant *Staphylococcus aureus* (CA-MRSA) infections are increasingly reported among gay, bisexual, and other men who have sex with men (GBMSM) engaging in chemsex, yet data remain limited. This ambispective cohort study conducted at Hospital Clínic of Barcelona (2018–2024) aimed to characterize CA-MRSA infections among GBMSM living with HIV. Methods: Epidemiological, behavioral, and clinical data were collected from medical records and standardized questionnaires. MRSA isolates from clinically indicated samples were identified by mass spectrometry and underwent antimicrobial susceptibility testing. Results: Among 446 participants, 62 (14%) were diagnosed with CA-MRSA, accounting for 109 infection episodes over a median follow-up of 3.21 years (IQR 1.39–5.05). All episodes involved skin and soft-tissue infections; 17% required hospitalization, 35% surgery, and 51% received non-active empirical antibiotics. Recurrent infections occurred in 32% of participants, and detectable HIV viral load was present in 28% of episodes. High levels of polydrug use were reported. Resistance to clindamycin (33%), cotrimoxazole (23%), and mupirocin (96%) was observed. Decolonization was attempted in 65% of participants. Conclusions: CA-MRSA infections were frequent, with substantial recurrence and antimicrobial resistance. These findings highlight challenges for infection control and antimicrobial stewardship and support further evaluation of preventive and decolonization strategies in this population.

## 1. Introduction

Community-associated methicillin-resistant *Staphylococcus aureus* (CA-MRSA) can cause a range of different clinical manifestations, from mild skin infections to severe invasive diseases. It often spreads in groups with close physical contact, such as children, prisoners, athletes, military personnel, and gay, bisexual, and other men who have sex with men (GBMSM) [1].

GBMSM are at increased risk of CA-MRSA due to overlapping clinical, treatment-related, and behavioral factors. Higher HIV incidence may impair immune response [2]. Additionally, frequent antimicrobial use in people living with HIV (PLHIV) to treat sexually transmitted infections (STIs) may promote resistance and the emergence of resistant strains, as highlighted in a systematic review and meta-analysis [3]. Risk factors for colonization include behaviors such as poor hygiene, drug use, incarceration, multiple sexual partners, unprotected anal sex, and overcrowded living. Thus, being GBMSM is not a risk factor per se; rather, specific high-risk behaviors increase vulnerability to CA-MRSA [1,3]. CA-MRSA is not classified as an STI, yet it may behave as one in specific contexts, as transmission can occur through close sexual contact.

The term chemsex refers to the practice among GBMSM of intentionally using specific drugs before or during sex to prolong, enhance, or facilitate the experience. This phenomenon has been described globally and has increased in recent years, especially in social and sexual settings [4,5,6]. Chemsex is linked to higher rates of HIV and STI transmission due to high-risk sexual behaviors [4]. The expansion of digital technologies, including internet-based platforms and smartphone applications, has facilitated new patterns of sexual networking and the spread of chemsex practices [7]. In parallel, the introduction of HIV pre-exposure prophylaxis (PrEP) and the growing prevalence of chemsex may be changing infection transmission patterns, highlighting the need for programmatic monitoring and targeted prevention strategies [8].

Beyond direct implications for prevention and clinical outcomes, CA-MRSA infections among GBMSM who engage in chemsex intersect with several critical public and global health domains, including antimicrobial resistance (AMR), emerging infectious threats within key populations [9], and implications for achieving Universal Health Coverage (UHC) [10].

The HIV research group at Hospital Clínic of Barcelona (HCB) was a pioneer in identifying and describing the rise of CA-MRSA cases in this specific population. De la Mora et al. found an 8% prevalence of CA-MRSA infections among PLHIV engaging in chemsex between 2018 and 2022, and identified high clonality among isolates, suggesting a shared transmission route [11]. These findings highlight the elevated risk in this population and underscore the need to consider CA-MRSA in relevant clinical presentations to ensure timely and appropriate treatment.

This study provides an updated overview of CA-MRSA infections within the HCB cohort, extending the observation period from February 2018 through June 2024, and expands on data previously reported by our group [12]. It describes the clinical, microbiological, and epidemiological characteristics of these infections.

Secondary objectives include examining sociodemographic factors, HIV-related parameters, antimicrobial resistance profiles, treatments administered, and decolonization strategies, as well as patterns of substance use and sexual behaviors, and reflecting on implications for strengthening infection management and rational antibiotic use within health services for this vulnerable group.

## 2. Materials and Methods

This ambispective longitudinal study was conducted between February 2018 and June 2024 at HCB. The center follows a prospective cohort of 446 PLHIV who engage in chemsex, derived from a pilot study [12] within the broader cohort of over 6500 PLHIV.

The study was carried out in accordance with the principles of the 2024 Declaration of Helsinki and was approved by the Institutional Review Board of HCB (HCB/2024/0513). All participants provided written informed consent.

Eligible participants consisted of GBMSM living with HIV included in the cohort of chemsex users who presented a probable CA-MRSA infection defined as (1) CA-MRSA isolation from clinical samples or (2) clinical presentation compatible with CA-MRSA infection without microbiological confirmation, but with a positive nasal, axillary, or perineal swab during the episode. Cases were identified in HIV outpatient clinics, the emergency department, or within the first 72 h of hospital admission, when applicable. Exclusion criteria included lack of confirmed MRSA isolation, discontinuation of chemsex practices, refusal to provide informed consent, or loss to follow-up preventing consent collection.

Methicillin-resistant *Staphylococcus aureus* was identified through isolation on agar plates and subsequent analysis using MALDI-ToF mass spectrometry (Bruker Daltonics, Bremen, Germany). Antimicrobial susceptibility was determined by disk diffusion on agar plates and broth microdilution using the Phoenix system (Becton Dickinson, Franklin Lakes, NJ, USA), following EUCAST guidelines [13].

Demographic data, including age, education level, region of origin according to the Joint United Nations Programme on HIV/AIDS (UNAIDS) classification [14], and country; HIV-related clinical and biological information (including CD4 cell count and HIV viral load); details on other STIs, patterns of drug use, and sexual behaviors over the preceding three months were collected retrospectively from medical records and prospectively using standardized surveys. Clinical, microbiological, and healthcare-associated characteristics, treatment, and infection outcomes were also recorded.

Follow-up time was defined as the interval from a participant’s first inclusion in a chemsex-related study within the cohort to the end of follow-up (June 2024), loss to follow-up, or death, as applicable, and was reported descriptively.

CA-MRSA-specific data were collected using an electronic case report form implemented in the REDCap system [15,16], hosted at HCB. The platform also enabled a self-administered electronic survey for chemsex participants. Sociodemographic information, HIV-related characteristics, and routine laboratory parameters were retrieved from the electronic medical record of the HIV Unit.

### Statistical Methods

Qualitative variables are described using absolute frequencies and percentages, calculated based on valid responses. Quantitative variables are presented as median and interquartile range (IQR). Analyses were conducted using available data for each variable; denominators therefore vary across analyses due to missing information. Baseline prevalence was calculated as the number of participants with CA-MRSA infections at study entry divided by the total number of participants. Cumulative incidence during the follow-up period was estimated as the number of new CA-MRSA cases diagnosed during follow-up divided by the total number of participants in the cohort. Both proportions are reported as percentages with their respective exact 95% confidence intervals (95% CI). Statistical analyses were performed using Stata version 18 (StataCorp LLC, College Station, TX, USA).

## 3. Results

Among the cohort of 446 PLHIV who engage in chemsex, 62 participants (14%) were identified with CA-MRSA infections during the study period, accounting for a total of 109 episodes. Table 1 provides an overview of the study population, CA-MRSA infection episodes, and diagnostic classification. Of these individuals, 16 (26%) had experienced at least one episode prior to or at the time of enrollment, 39 (63%) developed the infection during follow-up, and 7 (11%) had episodes both before and during the study period. The median follow-up time was 3.21 years (IQR 1.39–5.05).

More than two thirds of participants (71%) were born outside Spain, most frequently in Peru, Argentina, Brazil, and Venezuela. Over half of the cohort originated from Latin America and the Caribbean (LAC; 53%), followed by Western and Central Europe and North America (WCENA; 42%). Smaller proportions were born in the Middle East and North Africa (MENA; 3%) and in Asia and the Pacific (AP; 2%) [15].

The median CD4 lymphocyte count was 718 cells/mm^3^ (IQR 580;892). All participants were receiving antiretroviral therapy (ART); however, a detectable viral load was observed in 30 episodes (28%) with a median viral load of 395 cp/mL (IQR 65;15,000), and HIV-Hepatitis C virus (HCV) coinfection was documented in 16% of episodes (17/108). More sociodemographic and HIV-related clinical characteristics are detailed in Table 2.

**Figure 1:**
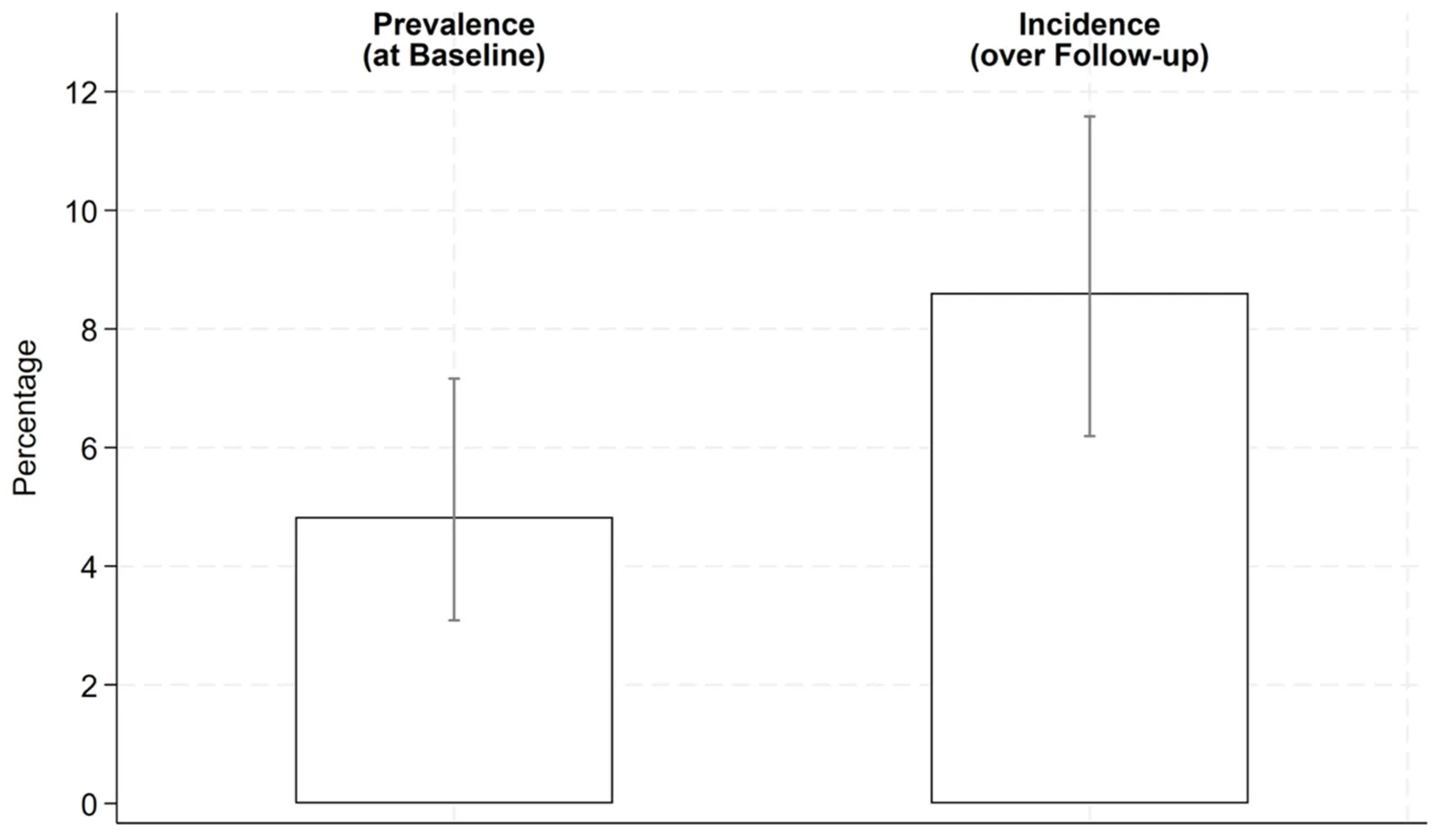
Baseline prevalence (**left**) and incidence over follow-up (**right**) of community-associated methicillin-resistant *Staphylococcus aureus* (CA-MRSA) infection episodes among 446 participants, expressed as percentages with 95% confidence intervals.

As illustrated in Figure 1, baseline prevalence of CA-MRSA infection reached 5% (95% CI: 3.3–7.6), while the cumulative incidence during follow-up was 9% (95% CI: 6.6–12.4). Overall, the risk of developing at least one CA-MRSA infection episode (either prior to or during the follow-up period) was 14% (95% CI: 10.8–17.5).

**Figure 2:**
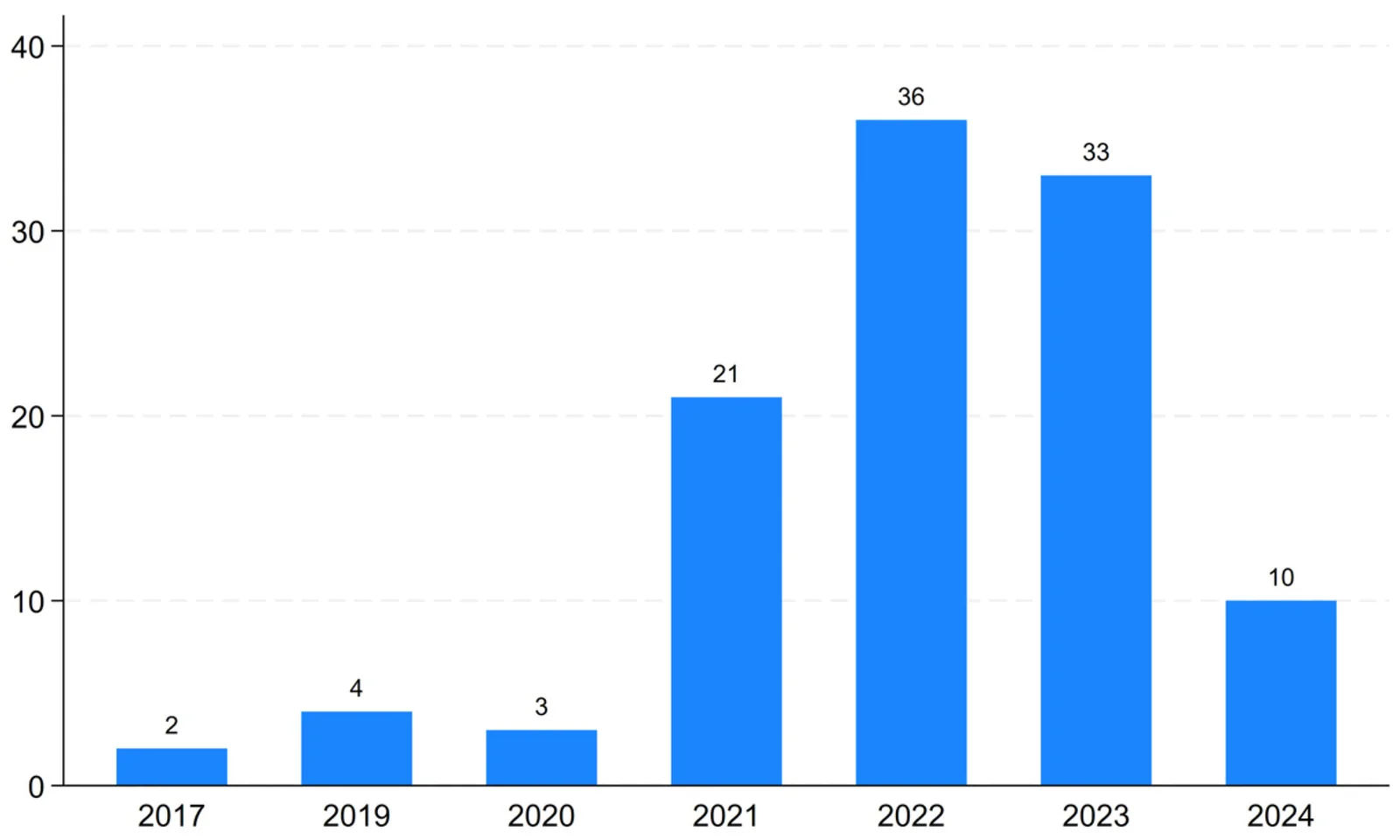
Annual number of community-associated methicillin-resistant *Staphylococcus aureus* (CA-MRSA) infection episodes among the study cohort of people living with HIV engaging in chemsex, 2017–2024. Data for 2024 include episodes diagnosed from January through June 2024.

The annual number of CA-MRSA episodes increased over the study period, with a peak observed in 2022–2023; data for 2024 are partial as follow-up ended in June (Figure 2).

All variables were assessed within the 12 months preceding each episode. Prior antibiotic use was reported in 91% of episodes (95/104). Prior hospitalization was recorded in 12% (12/102), liver disease in 11% (12/108), and the presence of indwelling catheters or permanent percutaneous devices in 9% (9/102). Surgical interventions were recorded in 5% of episodes (5/102), diabetes mellitus in 4% (4/108), and cotrimoxazole prophylaxis in 2% (2/109). No cases had a history of dialysis (0/107).

All MRSA episodes corresponded to skin and soft tissue infections (SSTIs), primarily abscesses (73% [80/109]) and cellulitis (41% [45/109]); both conditions co-occurred in 67% of those cellulitis episodes (30/45). The infections were predominantly located in the groin/gluteal/perineal area (39% [43/109]) and lower limbs (40% [44/109]). MRSA was directly isolated from the infection site in 93% of cases (87/94): the remaining 7% were diagnosed clinically, with negative local swabs but positive colonization swabs (6 nasal and 1 perineal).

A total of 73 nasal swabs were performed, yielding positive colonization results in 55% (40/73). Among cellulitis cases with microbiological data (*n* = 34), 59% (20/34) tested positive, of which 95% (19/20) were nasal.

Hospitalization was required in 17% of episodes (18/108), and 32% of patients (20/62) experienced multiple episodes during the study period; notably, 19% (12/62) experienced ≥3 episodes. Positive blood cultures were identified in 8% of cases (9/108), mainly bacteremia (78% [7/9]); no cases of endocarditis or prosthetic infection were observed.

In 51% of episodes (55/107), initial empirical antibiotic treatment was inactive; the initial empirical antibiotic did not cover MRSA according to susceptibility testing—most commonly amoxicillin/clavulanic acid (51% [22/43])—and was subsequently adjusted according to the antibiogram results when available. Linezolid was the most prescribed active antibiotic (56%), followed by clindamycin (35%) and cotrimoxazole (12%). Among isolates with susceptibility testing, universal resistance was observed to ciprofloxacin (17/17) and penicillin (84/84), with high resistance rates to levofloxacin (99%), mupirocin (96%), and erythromycin (95%). Resistance to clindamycin was observed in 33% (28/85) of isolates. All isolates were sensitive to linezolid and vancomycin (83/83 for both).

Drainage or surgical intervention was required in 35% of episodes. Decolonization strategies were applied in 65% of participants with available data (39/60), primarily through chlorhexidine gel body washes (85% [33/39]) and intranasal mupirocin (44% [17/39]). Follow-up nasal swabs were conducted in 21 participants, demonstrating successful decolonization in 71% (15/21). Detailed characteristics of CA-MRSA infections are presented in Table 3.

Among episodes with available information on previous STIs (*n* = 66), 79% (52/66) had a prior STI documented before the MRSA infection, with syphilis being the most prevalent (96% [46/48]). Participants reported having a steady partner in 20% of cases (17/86), and among them, 82% (14/17) indicated being in an open relationship. Risky sexual behaviors within the previous three months were reported in 92% of episodes (83/90), and in 91% (73/80) of those with ≥3 partners.

Regarding substance use, GHB/GBL was the most frequently consumed drug in 92% (88/96) of episodes with available data, followed by methamphetamine use in 84% (81/96). Although 69% of episodes (61/89) denied concurrent alcohol use with other drugs, 95% of episodes (90/95) reported polydrug use, and 52% (49/95) indicated using ≥3 substances. Oral administration was the predominant route, 95% (89/94), while injection drug use (slamming) was reported in 56% of episodes (32/57). The most common frequency of use was monthly, reported in 46% of episodes (45/97).

In 42% of episodes (31/73), respondents stated they did not remember the last time they had sex without substance use. Similarly, 42% (30/72) of episodes reported not having engaged in sober sex in the past six months. Additionally, in 60% of episodes (56/93), participants expressed a perceived need for help, and among them, 89% (50/56) reported specific concerns regarding their substance use in sexual contexts. Comprehensive data on substance use and sexual behaviors are detailed in Table 4.

## 4. Discussion

This study represents one of the largest cohort descriptions to date of CA-MRSA episodes among PLHIV who engage in chemsex worldwide. It expands on 25 cases previously reported in Barcelona [11]. Earlier studies from the United States reported high rates of colonization or infection in GBMSM, PLHIV, and people using drugs [17], including a colonization prevalence of 13% in HIV-infected outpatients [1]. In contrast, data from Spain in 2015 showed a much lower colonization prevalence (1–2%) [18], suggesting limited relevance at the time. However, those studies predate the widespread adoption of chemsex practices and PrEP, underscoring the need to reassess current risks in this evolving international context.

There is growing evidence that the rise of PrEP use and chemsex among GBMSM may be contributing to higher rates of classical STIs such as gonorrhea, chlamydia, and syphilis, while also facilitating the emergence of new pathogens like CA-MRSA [7,8]. Shared behavioral and network factors that drive STI transmission may also promote the spread of CA-MRSA, particularly among individuals engaging in high-risk practices like chemsex. These findings underscore the vulnerability of this population and the need for targeted clinical and preventive approaches.

Although all participants were on ART and had high median CD4 counts, nearly one-third had detectable viral loads at the time of infection, which may reflect suboptimal adherence or transient viral blips related to acute infection. Uncontrolled HIV viremia is a known risk factor for CA-MRSA infections and recurrences [19], and literature suggests that chemsex may impair virological control [6,12], increasing vulnerability to infections such as CA-MRSA. However, as this study was based on a clinical cohort without a comparison group, it was not designed to distinguish the respective contributions of HIV infection, behavioral factors, sexual networks, and repeated antimicrobial exposure to the occurrence of CA-MRSA infections.

The most frequent healthcare-associated characteristic observed was prior antibiotic use in the year prior to MRSA infection, likely linked to previous STIs and consequent antimicrobial exposure. Classic hospital-associated MRSA risk factors, such as dialysis, prolonged hospitalizations, surgical interventions, or invasive devices [20,21], were largely absent, supporting the community origin of the infections in this cohort.

All participants presented with skin and soft tissue infections, primarily located in the inguinal, gluteal, and perineal areas. This infection pattern aligns with the most common clinical presentation of MRSA in both the general population and among PLHIV [7,19,22,23,24], while the anatomical distribution is consistent with reports in GBMSM, where previous studies have suggested transmission through close sexual contact [17]. However, as this clinical cohort did not include molecular typing, our findings cannot confirm transmission pathways, and the possibility of sexual transmission should therefore be interpreted with caution.

Nasal swabs were often negative despite positive MRSA isolation from skin lesions, suggesting that colonization in this population may occur outside the nasal mucosa [18], likely due to sexual transmission. Therefore, in suspected cases of CA-MRSA infection (particularly in cellulitis without exudate), it is recommended to expand sample collection to alternative sites such as the inguinal or perianal regions, in addition to the standard nasal swab.

Over one-third of participants had recurrent infections, indicating limited effectiveness of current decolonization strategies. Even when standard nasal mupirocin-based protocols were properly followed, and decolonization was successful in over 70% of follow-up cases, recurrences were common, likely due to ongoing high-risk behavior and close contact with colonized individuals.

Nasal decolonization standard guidelines include mupirocin. Published studies generally report substantially lower mupirocin resistance rates among MRSA isolates [25,26] than those observed in our cohort. Our study found a 96% resistance rate to this antibiotic among isolates, significantly limiting its effectiveness. Although previous mupirocin exposure was not systematically assessed, mupirocin was frequently used for decolonization in this cohort and may partly explain the high resistance rate observed. Fusidic acid may be considered as an alternative; however, evidence supporting its efficacy in MRSA decolonization is scarce. Moreover, recent European data report the emergence and spread of mupirocin-resistant variants of the European epidemic fusidic acid-resistant S. aureus clone, further highlighting the growing challenge of antimicrobial resistance in this context [27]. These findings suggest that current decolonization guidelines may need to be updated to reflect evolving resistance patterns and to strengthen quality of care and rational antibiotic use within antimicrobial resistance agendas. Further studies evaluating fusidic acid-based decolonization regimens are warranted and are currently underway within our research group.

Although a standardized decolonization protocol exists at the hospital, the diagnosis across various healthcare settings, both inside and outside the hospital, results in inconsistent implementation, follow-up, and evaluation of efficacy. Documentation in medical records is frequently incomplete, making it difficult to determine whether standard decolonization regimens were prescribed and whether post-treatment assessments were performed. This variability hampers a unified approach among care providers and complicates continuity of follow-up. Furthermore, low adherence to scheduled visits limits ongoing monitoring of colonization status. Given that these individuals do not form a closed group but share persistent exposures, it is reasonable to question whether a single decolonization protocol is sufficient to prevent recurrences. Consequently, prolonged or individualized decolonization strategies, as proposed for other populations, should be explored [28].

More than half of participants received inappropriate initial antibiotic treatment, highlighting the need to raise awareness among frontline healthcare providers to improve early recognition and management of CA-MRSA infections in chemsex users.

Regarding resistance profiles, resistance rates were observed at 33% for clindamycin, 23% for cotrimoxazole, and 14% for tetracycline, limiting the available options for oral treatment in a non-hospital setting. A total of 91% of individuals had taken antibiotics prior to the CA-MRSA episodes, which likely contributed to the selection of resistant strains.

Regarding sexual behaviors, participants reported high-risk practices as well as a high prevalence of STIs. These circumstances have been widely described in the literature and are associated with an increased risk of CA-MRSA colonization and infection [7,22,23,24], likely due to close physical contact, microlesions in the skin and mucous membranes, and repeated exposure to antimicrobial treatments.

Nearly all participants reported using GHB/GBL, followed by methamphetamine, with polydrug use being reported almost universally. Non-injectable methamphetamine use has been identified as a risk factor for MRSA-related skin and soft tissue infections [20], due to its association with skin-picking behaviors and disruption of the skin barrier. When injected (slamming), methamphetamine use may facilitate the direct inoculation of microorganisms through the skin [3,29]. In our cohort, almost half of the participants reported engaging in slamming, reinforcing their exposure to multiple risk pathways for acquiring CA-MRSA.

From a global health perspective, the observed temporal increase in CA-MRSA episodes underscores the need for strengthened surveillance and timely prevention strategies within key populations. Understanding behavioral patterns surrounding substance use and sexual practices is essential for designing effective prevention and care strategies among GBMSM engaged in chemsex. More than half of participants in this cohort were born in LAC, highlighting the role of migration in shaping vulnerability to CA-MRSA. Migration-related factors, including social marginalization, barriers to access and continuity of care, and care across multiple health systems, may amplify the risk of infection and antimicrobial resistance, underscoring the need for health services responsive to increasingly diverse and transnational patient populations. The high rates of antimicrobial resistance observed in this cohort underscore the need for robust stewardship and innovation to safeguard the effectiveness of essential treatments, aligning with the World Health Organization (WHO) Global Action Plan on AMR and broader efforts toward UHC [10]. Addressing these infections requires a holistic approach that considers social determinants and structural vulnerabilities, as emphasized in the Sustainable Development Goals, to ensure equitable access to care for marginalized and migrant populations [30]. Integration of sexual health, substance use, and infectious disease services is vital for meeting the complex needs of affected individuals and reducing health disparities, in line with UNAIDS and the WHO strategies for key populations [9,31]. In this cohort, fewer than half of the participants recalled sober sex in the past six months, and in nearly 90% of the episodes, they expressed concern about their substance use in sexual contexts and a perceived need for help. These findings are consistent with previous studies that highlight a high level of concern and interest in self-regulation strategies within this population [32], and underscore the importance of developing tailored, integrated interventions that address both sexual health and substance use.

One limitation of this study is the absence of a comparison group, which precludes the identification of risk factors and limits causal inference regarding the study findings. In addition, it was not possible to assess all CA-MRSA infections among individuals who engage in chemsex, as not all eligible participants consented to participate. Self-administered questionnaires also led to frequent omissions, reducing data completeness. Some MRSA episodes might have gone unconfirmed microbiologically because of false-negative cultures, prior antibiotic use, or inadequate or indirect sampling. Moreover, the study was conducted at a single center in a specific population attending a referral hospital in a cosmopolitan city, potentially limiting the generalizability of the findings. Multicenter studies in diverse settings are needed to validate and broaden these results. Unlike our previous study, which included molecular epidemiology data and identified that 85% of CA-MRSA isolates belonged to the same clone [11], this analysis does not include molecular typing. Therefore, transmission pathways and whether recurrent infections were caused by the same or different strains cannot be determined from the present analysis alone. Nevertheless, previous molecular epidemiology studies conducted in this population have suggested substantial clonal spread, and dedicated molecular investigations of isolates from this cohort are currently underway to further characterize transmission dynamics and resistance determinants. Finally, contact tracing could not be performed due to the characteristics of the study population, which hinder the implementation of such interventions.

## 5. Conclusions

CA-MRSA infections represent an important clinical challenge in this cohort of GBMSM living with HIV who engage in chemsex. In cases of skin and soft tissue infections within this group, clinicians should consider a high index of suspicion and confirm the diagnosis through microbiological testing. Management may benefit from preventive strategies and standardized protocols, including sample collection from inguinal or perianal areas in addition to nasal swabs, given the likely sexual route of transmission.

Given the persistence of high-risk behaviors and frequent recurrences, prolonged or repeated decolonization regimens may be considered in preference to single-course protocols and adjusted to the resistance profile. Future research is needed to evaluate decolonization strategies tailored to PLHIV who engage in chemsex, aiming to reduce CA-MRSA recurrence and improve quality of care through strengthened antimicrobial stewardship. Finally, strengthening surveillance and evidence generation on emerging infectious threats among GBMSM living with HIV and engaging in chemsex, as well as among migrant and other at-risk populations, may support timely public health responses and the development of more targeted interventions at local, national, and global levels.

## Figures and Tables

**Table 1:** Overview of study population, CA-MRSA infection episodes, and diagnostic classification.

Level of Analysis	Measure	*n/N* (%)
Participant	Participants included in the study	446
	Participants with ≥1 CA-MRSA infection	62/446 (14%)
	Participants with recurrent CA-MRSA infection	20/62 (32%)
Episode	Total CA-MRSA infection episodes	109
	Episodes with diagnostic classification available	94/109 (86%)
	MRSA directly isolated from the infection site	87/94 (93%)
	Clinically diagnosed episodes	7/94 (7%)

CA-MRSA: community-associated methicillin-resistant *Staphylococcus aureus*; MRSA: methicillin-resistant *Staphylococcus aureus*. *N*: total number of participants included in the study; *n*: number of participants or infection episodes, as appropriate.

**Table 2:** Sociodemographic and HIV-related clinical characteristics.

**At Participant Level**		
**Demographic Characteristics**		
**Variable**		**Descriptive Statistics.** **Median (IQR)**
Age, years (*N* = 62)		42 (36;48)
		***n* (%)**
Region of origin (*N* = 62)	WCENA	26 (42%)
	Spain	18 (29%)
	Italy	3 (5%)
	France	2 (3%)
	United Kingdom	2 (3%)
	Germany	1 (2%)
	LAC	33 (53%)
	Peru	6 (10%)
	Argentina	5 (8%)
	Brazil	5 (8%)
	Venezuela	5 (8%)
	Colombia	3 (5%)
	Cuba	3 (5%)
	Chile	2 (3%)
	Ecuador	2 (3%)
	Costa Rica	1 (2%)
	Dominican Republic	1 (2%)
	MENA (Morocco)	2 (3%)
	AP (Philippines)	1 (2%)
Educational level (*N* = 53)	Primary incomplete	2 (4%)
	Primary complete	2 (4%)
	Secondary	26 (49%)
	University	23 (43%)
**At CA-MRSA episode level**		
**Serological data**		
**Serology (positive test result)**		***n* (%)**
HBsAg (*N* = 108)		4 (4%)
Anti-HBs (*N* = 108)		95 (88%)
Anti-HBc (*N* = 108)		31 (29%)
IgG anti-VHA (*N* = 108)		98 (91%)
Nontreponemal tests, VDRL * (*N* = 108)		82 (76%)
IgG anti-VHC (*N* = 108)		17 (16%)
ARN VHC (*N* = 17)		3 (18%)
**STD data**		
STD detected by PCR (positive test result) **		***n* (%)**
*Chlamydia trachomatis* (*N* = 106)		6 (6%)
*Neisseria gonorrhoeae* (*N* = 106)		27 (25%)
*Mycoplasma genitalium* (*N* = 33)		7 (21%)
**HIV data**		
Transmission route (*N* = 61)	GBMSM—IDU	2 (3%)
	GBMSM—No IDU	59 (97%)
**Lymphocyte subpopulations**		**Median (IQR)**
Nadir CD4+ lymphocyte count (cells/mm^3^) (*N* = 109)		379 (291;530)
CD4+ T lymphocyte count (cells/mm^3^) (*N* = 109)		718 (580;892)
CD8+ T lymphocyte count (cells/mm^3^) (*N* = 109)		850 (583;1093)
CD4/CD8 ratio (*N* = 109)		0.9 (0.7;1.2)
**Viral load (VL)**	**VL**	***n* (%)**
VL HIV-1 PCR (*N* = 109)	Detectable	30 (28%)
	Undetectable	79 (72%)
Detectable HIV VL (cp/mL) (*N* = 30)	**Median (IQR)**	
	395 (65;15,000)	
**ART (N = 107)**	**Treatment**	***n* (%)**
	NNRTI	19 (18%)
	PI	11 (10%)
	INSTI	83 (78%)

IQR: interquartile range, WCENA: Western and Central Europe and North America, LAC: Latin America and the Caribbean, MENA: Middle East and North Africa, AP: Asia Pacific, HBsAg: hepatitis B surface antigen, Anti-HBs: hepatitis B surface antibody, Anti-HBc: hepatitis B core antibody, IgG anti-HAV: hepatitis A virus IgG antibody, IgG anti-HCV: hepatitis C virus IgG antibody, STDs: sexually transmitted diseases, GBMSM: gay, bisexual and other men who have sex with men, IDU: injecting drug use, VL: viral load, NNRTI: nonnucleoside reverse transcriptase inhibitors, PI: protease inhibitors, INSTI: integrase strand transfer inhibitors. * Positive nontreponemal tests refer to an individual’s past clinical history. ** STDs detected by PCR (overall locations: urethra, pharynx and rectum) at baseline or during follow-up of the chemsex cohort.

**Table 3:** MRSA characterization.

	***n* (%)**
**Healthcare-associated characteristics** (past 12 months)	
Antibiotic use (*N* = 104)	95 (91%)
Dialysis (*N* = 107)	0
Surgery (*N* = 102)	5 (5%)
Hospitalization * (*N* = 102)	12 (12%)
Permanent catheters or percutaneous devices (*N* = 102)	9 (9%)
Cotrimoxazole prophylaxis (*N* = 109)	2 (2%)
Diabetes (*N* = 108)	4 (4%)
Liver disease (*N* = 108)	12 (11%)
**Clinical characteristics**	
SSTI (*N* = 109)	109 (100%)
Infections associated with SSTIs (*N* = 108)	9 (8%)
Bacteremia (*N* = 9)	7 (78%)
Pneumonia (*N* = 9)	1 (11%)
Osteoarticular infections (*N* = 9)	1 (11%)
**SSTI clinical presentation**	
Abscess (*N* = 109)	80 (73%)
Cellulitis ** (*N* = 109)	45 (41%)
Folliculitis (*N* = 109)	9 (8%)
Furunculosis (*N* = 109)	16 (15%)
**SSTI localization**	
Head/neck (*N* = 109)	17 (16%)
Trunk (*N* = 109)	14 (13%)
Upper extremities (*N* = 109)	14 (13%)
Groin/buttocks/perineum (*N* = 109)	43 (39%)
Lower extremities (*N* = 109)	44 (40%)
**Other clinical characteristics**	
Hospitalization (*N* = 108)	18 (17%)
Recurrence *** (*N* = 62)	20 (32%)
Colonization (*N* = 73)	40 (55%)
Nasal colonization (*N* = 40)	39 (98%)
Perineum colonization (*N* = 40)	1 (2%)
Isolation (*N* = 94)	87 (93%)
During hospitalization (<72 h) (*N* = 82)	5 (6%)
During outpatient visit (*N* = 82)	50 (61%)
During emergency department visit (*N* = 82)	27 (33%)
**Treatment**	
Non-active empirical antibiotics (*N* = 107)	55 (51%)
Incision and drainage or surgery (*N* = 109)	38 (35%)
No treatment (*N* = 109)	3 (3%)
Antibiotics (*N* = 109)	106 (97%)
Ceftaroline (*N* = 34)	1 (3%)
Clindamycin (*N* = 34)	12 (35%)
Cotrimoxazole (*N* = 34)	4 (12%)
Dalbavancin (*N* = 34)	1 (3%)
Daptomycin (*N* = 34)	2 (6%)
Linezolid/Tedizolid (*N* = 34)	19 (56%)
Teicoplanin (*N* = 34)	1 (3%)
	***n* (%)**
Decolonization (*N* = 60)	39 (65%)
Nasal Mupirocin (*N* = 39)	17 (44%)
Chlorhexidine mouthwash (*N* = 39)	3 (8%)
Baths with Chlorhexidine gel (*N* = 39)	33 (85%)
Betadine^®^ shampoo (*N* = 39)	1 (3%)
Fusidic acid cream (*N* = 33)	4 (12%)
Effective decolonization (*N* = 21)	15 (71%)
**Antibiotic resistance of MRSA isolates**	***n* (%)**
From the site of infection ****	
Ciprofloxacin (*N* = 17)	17 (100%)
Clindamicyn (*N* = 85)	28 (33%)
Cotrimoxazole (*N* = 84)	19 (23%)
Erythromycin (*N* = 82)	78 (95%)
Gentamicin (*N* = 85)	5 (6%)
Levofloxacin (*N* = 83)	82 (99%)
Linezolid (*N* = 83)	0
Mupirocin (*N* = 48)	46 (96%)
Oxacillin (*N* = 87)	87 (100%)
Penicillin (*N* = 84)	84 (100%)
Teicoplanin (*N* = 51)	1 (2%)
Tobramycin (*N* = 50)	11 (22%)
Tetracycline (*N* = 7)	1 (14%)
Vancomycin (*N* = 83)	0

SSTI: skin and soft tissue infection. * Hospitalization or long-term care facility admission, at least a 1-night stay. ** It can coexist with other SSTIs. *** More than one episode during the follow-up period, after the first diagnosis. **** Antimicrobial susceptibility testing was performed as part of routine clinical practice. Consequently, not all antimicrobial agents were tested for every isolate, and the number of isolates evaluated varied according to the antibiotic tested.

**Table 4:** Chemsex-related characteristics.

**Clinical History**		***n* (%)**
Previous STI diagnosis (*N* = 66)		52 (79%)
	Syphilis (*N* = 48)	46 (96%)
	*Neisseria gonorrhoeae* (*N* = 52)	31 (60%)
	*Chlamydia tracomatis* (*N* = 52)	28 (54%)
	Crabs (*N* = 52)	7 (13%)
	Proctitis (*N* = 27)	23 (85%)
	Scabies (*N* = 30)	26 (87%)
	Urethritis (*N* = 34)	30 (88%)
	Chancroid (*N* = 52)	2 (4%)
**Sexual practices**		***n* (%)**
Oral sex (*N* = 96)		95 (99%)
	With condom (*N* = 95)	2 (2%)
	Sometimes condom (*N* = 95)	13 (14%)
Anal sex (*N* = 96)		93 (97%)
	With condom (*N* = 91)	4 (4%)
	Sometimes condom (*N* = 91)	28 (31%)
Double penetration (*N* = 93)		42 (45%)
	Sometimes condom (*N* = 43)	5 (12%)
	No condom use (*N* = 43)	37 (86%)
**Partner type and sexual behavior**		***n* (%)**
Current partner (*N* = 86)		17 (20%)
Open relationship (*N* = 17)		14 (82%)
High-risk sex L3M (*N* = 90)		83 (92%)
Sexual partners L3M (*N* = 80)		
	<3	7 (9%)
	3–10	27 (34%)
	11–30	29 (36%)
	≥31	17 (21%)
**Drugs**		***n* (%)**
Use of substances to enhance sexual pleasure (*N* = 97)		97 (100%)
At least one in L6M (*N* = 76)		37 (49%)
Substance		
Cocaine (*N* = 96)		25 (26%)
Ketamine (*N* = 96)		28 (29%)
GHB/GBL (*N* = 96)		88 (92%)
Methamphetamine (*N* = 96)		81 (84%)
**Drugs**		***n* (%)**
Mephedrone (*N* = 96)		48 (50%)
Amphetamine (*N* = 96)		7 (7%)
Ectasis (pastillas) (*N* = 96)		19 (20%)
MDMA (*N* = 96)		19 (20%)
Poppers (*N* = 96)		62 (65%)
Sildenafil (*N* = 96)		71 (74%)
Cannabis (*N* = 96)		31 (32%)
Tusi (*N* = 96)		5 (5%)
Alfa-PVP, Flakka (*N* = 71)		8 (11%)
Bath salts (*N* = 71)		8 (11%)
Chloroethyl (*N* = 71)		14 (20%)
Caverject^®^ (*N* = 71)		13 (18%)
Synthetic cannabinoids (*N* = 71)		1 (1%)
Metaphedrone (*N* = 96)		10 (10%)
LSD (*N* = 71)		1 (1%)
Heroine (*N* = 71)		1 (1%)
Crack (*N* = 71)		7 (10%)
Tropicamide eye drops (*N* = 71)		10 (14%)
Atropine eye drops (*N* = 71)		6 (8%)
Concomitant alcohol use (*N* = 89)		
	Yes	4 (4%)
	Occasionally	24 (27%)
	No	61 (69%)
Number of drugs (*N* = 95)		
	1	5 (5%)
	2	41 (43%)
	≥3	49 (52%)
*Route of administration*		
Oral (*N* = 94)	Yes	80 (85%)
	Occasionally	9 (10%)
Inhaled (*N* = 88)	Yes	71 (81%)
	Occasionally	10 (11%)
Snorted (*N* = 70)	Yes	36 (51%)
	Occasionally	13 (19%)
Sublingual (*N* = 52)	Yes	6 (12%)
	Occasionally	1 (2%)
Rectal (*N* = 55)	Yes	4 (7%)
**Drugs**		***n* (%)**
Rectal (*N* = 55)	Occasionally	17 (31%)
Slamming (*N* = 57)	Yes	24 (42%)
	Occasionally	8 (14%)
Slamming: shares injection equipment (*N* = 27)	Yes	1 (4%)
	Occasionally	1 (4%)
Slamming: use of sterile equipment (*N* = 42)	Yes	28 (67%)
	Occasionally	3 (7%)
Frequency of use (*N* = 97)		
	Daily	6 (6%)
	Weekly	31 (32%)
	Monthly	45 (46%)
	<Monthly	15 (15%)
Remembers last time having sober sex (*N* = 73)	Yes	42 (58%)
	No	31 (42%)
Sober sex episodes L6M (*N* = 72)	Never/Rarely	30 (42%)
	At least once	29 (40%)
	Almost always/Always	13 (18%)

STD: sexual transmitted disease, HCV: hepatitis C virus, L3M: last 3 months, L6M: last 6 months.

## Data Availability

The data that support the findings of this study are available from the corresponding author upon reasonable request.

## Abbreviations

The following abbreviations are used in this manuscript:

CA-MRSA	Community-associated methicillin-resistant *Staphylococcus aureus*
GBMSM	Gay, bisexual, and other men who have sex with men
PLHIV	People living with HIV
STIs	Sexually transmitted infections
PrEP	HIV pre-exposure prophylaxis
AMR	Antimicrobial resistance
UHC	Universal Health Coverage
HCB	Hospital Clínic of Barcelona
IQR	Interquartile range
CI	Confidence interval
ART	Antiretroviral therapy
UNAIDS	The Joint United Nations Programme on HIV/AIDS
LAC	Latin America and the Caribbean
WCENA	Western and Central Europe and North America
MENA	Middle East and North Africa
AP	Asia and the Pacific
WHO	World Health Organization
